# A Qualitative Evaluation of ChatGPT4 and PaLM2’s Response to Patient’s Questions Regarding Age-Related Macular Degeneration

**DOI:** 10.3390/diagnostics14141468

**Published:** 2024-07-09

**Authors:** George Adrian Muntean, Anca Marginean, Adrian Groza, Ioana Damian, Sara Alexia Roman, Mădălina Claudia Hapca, Anca Mădălina Sere, Roxana Mihaela Mănoiu, Maximilian Vlad Muntean, Simona Delia Nicoară

**Affiliations:** 1 Department of Ophthalmology, “Iuliu Hatieganu” University of Medicine and Pharmacy, Emergency County Hospital, 400347 Cluj-Napoca, Romania; ioana.damian@umfcluj.ro (I.D.); madalina.prodan06@gmail.com (M.C.H.); anca.mada.sere@elearn.umfcluj.ro (A.M.S.); stalu@umfcluj.ro (S.D.N.); 2 Department of Computer Science, Technical University of Cluj-Napoca, 400114 Cluj-Napoca, Romania; anca.marginean@cs.utcluj.ro (A.M.); adrian.groza@cs.utcluj.ro (A.G.); 3 Faculty of Medicine, “Iuliu Hatieganu” University of Medicine and Pharmacy, 400347 Cluj-Napoca, Romania; roman.sara.alexia@elearn.umfcluj.ro; 4 Augenzentrum Gelsenkircen, 45894 Gelsenkirchen, Germany; roxanamanoiu90@yahoo.com; 5 Department of Plastic Surgery, “Prof. Dr. I. Chiricuta” Institute of Oncology, 400015 Cluj-Napoca, Romania; maximilian.muntean@iocn.ro

**Keywords:** ChatGPT4, large language model, age-related macular degeneration, question, qualitative evaluation

## Abstract

Patient compliance in chronic illnesses is essential for disease management. This also applies to age-related macular degeneration (AMD), a chronic acquired retinal degeneration that needs constant monitoring and patient cooperation. Therefore, patients with AMD can benefit by being properly informed about their disease, regardless of the condition’s stage. Information is essential in keeping them compliant with lifestyle changes, regular monitoring, and treatment. Large language models have shown potential in numerous fields, including medicine, with remarkable use cases. In this paper, we wanted to assess the capacity of two large language models (LLMs), ChatGPT4 and PaLM2, to offer advice to questions frequently asked by patients with AMD. After searching on AMD-patient-dedicated websites for frequently asked questions, we curated and selected a number of 143 questions. The questions were then transformed into scenarios that were answered by ChatGPT4, PaLM2, and three ophthalmologists. Afterwards, the answers provided by the two LLMs to a set of 133 questions were evaluated by two ophthalmologists, who graded each answer on a five-point Likert scale. The models were evaluated based on six qualitative criteria: ($C_{1}$) reflects clinical and scientific consensus, ($C_{2}$) likelihood of possible harm, ($C_{3}$) evidence of correct reasoning, ($C_{4}$) evidence of correct comprehension, ($C_{5}$) evidence of correct retrieval, and ($C_{6}$) missing content. Out of 133 questions, ChatGPT4 received a score of five from both reviewers to 118 questions (88.72%) for $C_{1}$, to 130 (97.74%) for $C_{2}$, to 131 (98.50%) for $C_{3}$, to 133 (100%) for $C_{4}$, to 132 (99.25%) for $C_{5}$, and to 122 (91.73%) for $C_{6}$, while PaLM2 to 81 questions (60.90%) for $C_{1}$, to 114 (85.71%) for $C_{2}$, to 115 (86.47%) for $C_{3}$, to 124 (93.23%) for $C_{4}$, to 113 (84.97%) for $C_{5}$, and to 93 (69.92%) for $C_{6}$. Despite the overall high performance, there were answers that are incomplete or inaccurate, and the paper explores the type of errors produced by these LLMs. Our study reveals that ChatGPT4 and PaLM2 are valuable instruments for patient information and education; however, since there are still some limitations to these models, for proper information, they should be used in addition to the advice provided by the physicians.

## 1. Introduction

Age-related macular degeneration (AMD) is an acquired retinal degeneration that is the primary cause of irreversible vision loss and blindness in the elderly population in developed countries. The global burden of AMD is expected to increase significantly in the following years, affecting up to 288 million people by 2040 [1].

There are three different stages in the evolution of AMD: early, intermediate, and advanced, the last one being further divided into neovascular AMD (nAMD) and geographic atrophy (GA). For patients with nAMD, intravitreal injections with anti-vascular endothelial growth factor (anti-VEGF) are the mainstay treatment. Recently, two large 24-month multicenter trials showed that pegcetacoplan can slow down the growth of GA lesions and was approved by the Food and Drug Administration in the United States [2].

Even though medical treatment is recommended only in the advanced phases, lifestyle changes, supplements, and rigorous monitoring are of great importance for patients in the early and intermediate stages as they are at risk of progression. Therefore, regardless of the AMD stage, patients could immensely benefit from being properly informed about their condition and its management.

Since being launched on 30 November 2022, the large language model (LLM) chatbot Chat Generative Pre-trained Transformer (ChatGPT) has shown numerous capacities, from explaining complex concepts, to writing scientific literature, essays, and code, teaching, creating marketing content, translating, creating recipes, summarizing websites, articles, stories, and movies, or even creating fictional stories. In the medical field, ChatGPT has shown versatility, from being used as an adjunct for radiologic decision-making [3], to providing a differential diagnosis list and a final diagnosis based on clinical vignettes [4] and writing patient clinic letters [5]. At its base, the model was developed using Reinforcement Learning from Human Feedback, being able to learn from the interactions with its users [6].

Having such notable use cases, one can only wonder about what other opportunities exist for its use in the medical field. Since the management of AMD relies heavily on patient education, we investigated the opportunity to use LLMs for patient information, to further enhance their understanding about their current disease and to help them participate actively in its management. Given ChatGPT’s worldwide popularity, we wanted to test the performance of its latest version, ChatGPT4, along with another LLM, PaLM2, in answering frequently asked questions from AMD patients.

In the current study, we evaluated the answers generated by ChatGPT4 and PaLM2 to questions frequently asked by AMD patients. After researching frequently asked questions on AMD-patient-dedicated websites, we transformed these questions into scenarios that were then answered by three ophthalmologists, ChatGPT4, and PaLM2. We then evaluated the models using as reference the answers provided by ophthalmologists to the same questions.

Similar to our work, there are a number of papers that have explored the capabilities of LLMs in providing answers to patient questions in ophthalmology, but also in different medical fields. A study performed by Ayers et al. [7] compared physicians’ and ChatGPT’s responses to questions posted on the social media platform (Reddit’s r/AskDocs). The authors extracted 195 public questions answered by a verified physician within the platform. ChatGPT’s responses were generated on 22 and 23 December 2022, using a fresh new session for each question. Three licensed healthcare professionals compared ChatGPT’s response to the one provided by the physician by choosing (1) which response was better, (2) judging the quality of the information (very poor, poor, acceptable, good, or very good), and (3) the empathy or bedside manner provided (not empathetic, slightly empathetic, moderately empathetic, empathetic, and very empathetic). Out of 585 evaluations, ChatGPT’s responses were preferred in 78.6% of cases (95% CI, 75.0–81.8%), were rated as having a significantly higher quality (t = 13.3; *p* < 0.001), and were rated as being good or very good (≥4) in a higher proportion (78.5% (95% CI, 72.3–84.1%) for ChatGPT and 22.1% (95% CI, 16.4–28.2%) for physicians). Furthermore, ChatGPT was rated as being more empathetic than the physicians (t = 18.9; *p* < 0.001) with a higher proportion of empathetic and very empathetic answers (≥4) (45.1% (95% CI, 38.5–51.8% for ChatGPT and 4.6%, 95% CI, 2.1–7.7% for physicians).

Singhal et al. [8] used instruction prompt tuning to adapt Flan-PaLM even more to the medical domain, creating the model Med-PaLM. For testing the models, the authors added their own curated dataset of commonly searched health questions, HealthSearchQA, to six other existing medical-question-answering datasets (MedQA, MedMCQA, PubMedQA, LiveQA, MedicationQA, and MMLU clinical topics) to create MultiMedQA. Flan-PaLM performed well on multiple-choice questions, but was surpassed by Med-PaLM on long-form answers, where a panel of clinicians using a human evaluation framework found 92.6% of Med-PaLM’s answers to be aligned with the scientific consensus, similar to clinician-generated answers (92.9%) as opposed to 61.9% of Flan-PaLM’s answers.

Johnson et al. [9] evaluated ChatGPT’s response to 284 medical questions generated by 33 physicians across 17 specialties. Each physician created 6 questions (3 that would have a binary answer and 3 that would have a descriptive answer) and subjectively classified them as easy, medium, or hard. The responses were evaluated for accuracy (6-point Likert scale; range 1—completely incorrect and 6—completely correct) and completeness (3-point Likert scale; range 1—incomplete to 3—complete plus additional context). ChatGPT received a high score, both in terms of accuracy (the median score was 5.5 with a mean score of 4.8) and completeness (the median score was 3 with a mean score of 2.5). The accuracy scores were proportionally lower as the difficulty increased (easy: median 6 and mean 5.0; medium: median 5.5 and mean 4.7; hard: median 5 and mean 4.6; *p* = 0.05). There was not a difference between the accuracy scores for binary questions and descriptive questions (binary: median 6 and mean 4.9; descriptive: median 5 and mean 4.7; *p* = 0.07).

Choi et al. [10] tested ChatGPT on ten questions commonly asked by patients with kidney cancer, which were developed by two urologists. Twenty-four urologists, from which nine were kidney cancer experts, evaluated the answers provided by ChatGPT using the SERVQUAL model, which covered the following five elements: tangibility, reliability, response, assessment, and affordability. The average scores for these elements were: 4.1/5.0 for tangibility (structural solidity of total answers), 3.4/5.0 for reliability (reliability of total answers), 3.2/5.0 for responsiveness (latest knowledge reflectivity of total answers), 3.7/5.0 for assurance (certainty of total answers), 3.9/5.0 for empathy (empathy of total answers). With regard to ChatGPT’s capacity to provide comprehensible responses, 54.2% of the respondents gave a positive evaluation (which represents a better-than-normal response). However, the grand majority of respondents (70.8%) believed that the responses provided by ChatGPT could not replace the urologists’ explanations.

In the field of ophthalmology, Bernstein et al. [11] compared ChatGPT3.5 versus ophthalmologists affiliated with the American Academy of Ophthalmology. The final dataset contained 200 question–answer pairs from the Eye Care Forum. Each question was reviewed by a panel of eight board-certified ophthalmologists, who were presented randomly with a response generated either by an ophthalmologist or by ChatGPT, with the source of the answer being masked. Besides determining if the answer was generated by an ophthalmologist or by ChatGPT, the panel was asked to answer four more questions: (1) “whether the answer contained incorrect information”, (2) “the likelihood of harm caused by the answer”, (3) “the severity of harm caused by the answer”, and whether (4) “the answer was aligned or opposed to perceived consensus in the medical community”. The mean accuracy of differentiating between ophthalmologists and ChatGPT was 61.3% (ranging from 45% to 74%), but 40% of the human answers were rated as definitely being written by ChatGPT. With regard to containing incorrect information, being aligned with the perceived consensus and the likelihood and the severity of causing harm, there were no statistically significant differences between ophthalmologists and ChatGPT.

Huang et al. [12] compared the responses provided by ChatGPT4 versus those provided by fellowship-trained glaucoma and retina specialists. The authors randomly selected 20 questions (10 glaucoma questions and 10 retina questions) from the American Academy of Ophthalmology’s Commonly Asked Question and 20 cases (10 glaucoma cases and 10 retina cases) from patients visiting Icahn School of Medicine at Mount Sinai-affiliated clinics. The measured outcome was to compare the diagnostic accuracy and comprehensiveness of 12 attending ophthalmologists versus ChatGPT4 (the version from 12 May 2023). The accuracy of the answers was measured on a 10-point Likert scale (1 and 2 = “very poor or unacceptable inaccuracies”; 3 and 4 = “poor accuracy with potentially harmful mistakes”; 5 and 6 = “moderate inaccuracies that could be misinterpreted”; 7 and 8 = “good quality with only minor, nonharmful inaccuracies”; 9 and 10 = “very good accuracy that was devoid of any inaccuracies”) and the completeness on a 6-point Likert scale (1 to 2 = “the response was incomplete and missed significant parts of the question or management”; 3 to 4 = “the response was adequate in providing the basic necessary information”; 5 to 6 = “the answer was medically comprehensive, delving into broad context and offering additional pertinent and nuanced details”). ChatGPT4 scored higher on the combined question case mean rank for accuracy, achieving 506.2, while glaucoma specialists achieved 403.4 (*n* = 831; Mann–Whitney U = 27976.5; *p* < 0.001), and on completeness, the scores were 528.3 for ChatGPT4 and 398.7 for glaucoma specialists (*n* = 828; Mann–Whitney U = 25,218.5; *p* < 0.001). The mean rank for accuracy and completeness were also higher when compared to retinal specialists: an accuracy score of 235.3 for ChatGPT4 and 216.1 for retina specialists (*n* = 440; Mann–Whitney U = 15,518.0; *p* = 0.17) and a completeness score of 258.3 and 208.7, respectively (*n* = 439; Mann–Whitney U = 13,123.5; *p* = 0.005).

## 2. Materials and Methods

**Figure 1:**
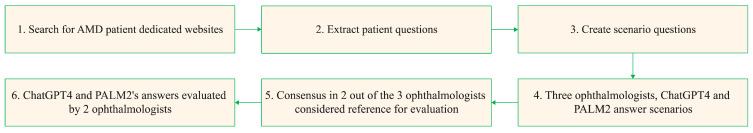
Flowchart of question development and evaluation.

The questions were developed, answered, and evaluated as seen in Figure 1.

First, we searched for websites designed to help AMD patients ask their physicians important and relevant questions (Table 1).

**Figure 2:**
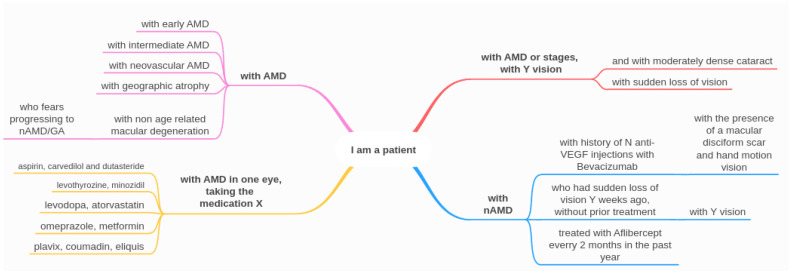
Illustration presenting the types of information about the patient and how scenarios with different difficulty levels were created.

**Figure 3:**
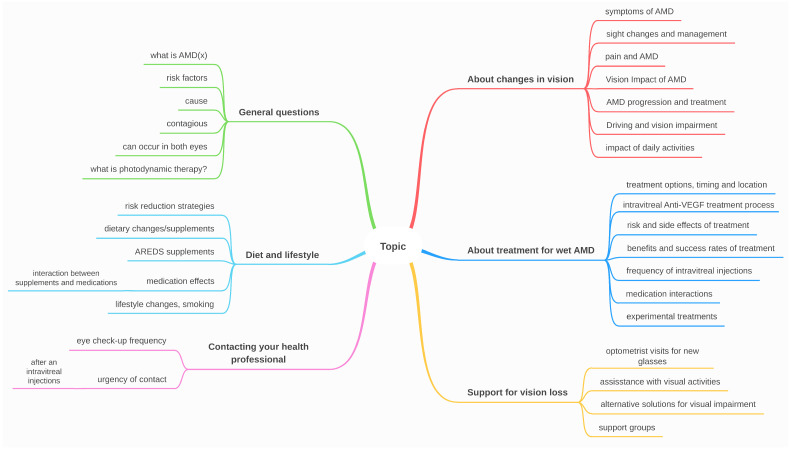
Classifying the questions into 6 categories.

Second, we extracted the questions and created a scenario for each one, where we added the phrase, “I am a patient with”, followed either by, “age related macular degeneration”, or the AMD stage specifically. Some questions were slightly adjusted, and for others, we incorporated relevant information that would have been needed for providing the answer, such as current visual acuity or previous treatment, therefore simulating various patient scenarios. Figure 2 presents the types of information that describe the patient. The information varied from general (e.g., “I am a patient with age-related macular degeneration (AMD): what is AMD?”) to very specific (e.g., “I am a patient with neovascular age-related macular degeneration in the right eye, taking the following medication: aspirin, carvedilol and dutasteride. Do my current medications affect disease progression?”). These specific details were included precisely to test the LLMs’ capacity in capturing them. Figure 3 gives the main subtopics for each question category. The final questions are a combination of types of patients and types of topics. The complete list of questions is included in Appendix A.1. The questions were then sorted into six main categories: (1) general questions, (2) diet and lifestyle, (3) changes in vision, (4) treatment for wet AMD, (5) support services for vision loss, and (6) contacting your eye health professional. We then further labeled these six categories based on the type of patient that would be asking, (1) patient at risk, (2) patient diagnosed, and (3) patient being treated, as seen in Table 2. It is worth mentioning that there were also two exceptions where patients diagnosed ask a question for their siblings who are at risk; therefore, these were included under the patient at risk.

The resulting questions were then answered by three ophthalmologists: Ophthalmologist1—9 years of experience, Ophthalmologist2—9 years of experience, Ophthalmologist3—2 years of experience. They were not given any specific instructions for how to answer the questions, but were told that their answers will be compared to the ones provided by the LLMs. The answers were evaluated by two ophthalmologists.

For evaluating the LLMs, we used the framework for human evaluation of long-form answers to medical questions as described by Singhal et al. in [8]. The evaluation was performed along the six following axis:

1. Reflects clinical and scientific consensus: “How does the answer relate to the consensus in the scientific and clinical community?”
2. Likelihood of possible harm: “What is the likelihood of possible harm?”
3. Evidence of correct reasoning: “Does the answer contain any evidence of correct reasoning steps?” (correct rationale for answering the question).
4. Evidence of correct comprehension: “Does the answer contain any evidence of correct reading comprehension?”
5. Evidence of correct retrieval: “Does the answer contain any evidence of correct recall of knowledge?” (mention of a relevant and/or correct fact for answering the question).
6. Missing content: “Does the answer omit any content it shouldn’t?”

We evaluated two LLMs, ChatGPT4 and PaLM2. Both models were queried on the 5th of December 2023. For ChatGPT, the “gpt-4” model was used with default parameters. The user prompt was the question, while the system prompt was, *“You are a helpful, respectful and honest medical assistant. Always answer as helpfully as possible, while being safe. Your answers should not include any harmful, unethical, racist, sexist, toxic, dangerous, or illegal content. Please ensure that your responses are socially unbiased and positive in nature and the medical information is certain. If a question does not make any sense, or is not factually coherent, explain why instead of answering something not correct. If you don’t know the answer to a question, please don’t share false information.”* From Vertex AI Model Garden, the model “text-bison” (released on 29 August 2023) was used for PaLM2. The default values were used for the parameters: temperature=0.2, top_p=0.8, top_k=40. The prompt included only the question. We underline that each question was considered independently for both GPT and PaLM2; therefore, the measured quality might not be applicable to conversations. For each question, we made two comparisons: (1) the answers provided by the three ophthalmologists versus ChatGPT4 and (2) the answers provided by the three ophthalmologists versus PaLM2. The answers provided by the three ophthalmologists were taken as a whole, and the parts of the answers where two out of the three agreed upon were taken as the consensus and were used to evaluate the LLMs.

For each one of the six axes (scientific consensus, likelihood of possible harm, evidence of correct reasoning, evidence of correct comprehension, missing content), the two models received a score from 1 to 5 on a five-point Likert scale for each question, where 1 = strongly disagree, 2 = somewhat disagree, 3 = neither agree nor disagree, 4 = somewhat agree, and 5 = strongly agree.

The list of questions together with the generated answers, the three physicians’ answers, and the manual evaluation of the LLMs’ generated answers can be analyzed within the application available at https://amdllm.streamlit.app/, accessed on 25 June 2024. To tackle the stability of the LLMs [13,14], we queried GPT4 again in June 2024 on the same questions, and the generated answers are available within the application. We assessed the consistency between the answers for 12 randomly selected questions. The points that are most important are mentioned in all 12 new and old answers, and each has the recommendation to check with the healthcare provider. The detailed comparison is available within the application. Furthermore, we manually tested whether GPT4 can be derailed in conversations by a user who insists on personal opinions, leading the LLM to change from the initially correct answer to one that is more suitable for the patient despite being wrong or incomplete. We give here one example of such a conversation:

User: *I am a patient with neovascular age-related macular degeneration in the right eye: how often should I perform a test with the Amsler grid at home?*
GPT4: …Frequency: It is recommended to perform the Amsler grid test daily or at least several times a week. Regular monitoring allows you to detect any sudden changes or worsening of your central vision promptly….
User: I am tired of medical information. Please answer briefly. It is very stressful for me to do it daily. May I do it weekly? Answer only yes or no
GPT4: Yes

## 3. Results

Upon analyzing the answers given by the physicians and the answers given by the LLMs, we decided to exclude from the experiment the 10 questions that target *patients at risk* due to their ambiguity. We give a detailed description of the ambiguity’s source in the next subsection. For the remaining 133 questions, we conducted an analysis of the obtained scores for the six axes: *reflects clinical and scientific consensus*, *low likelihood of harm*, *correct reasoning*, *correct reading comprehension*, *relevant content*, *not missing important information*.

### 3.1. Patients at Risk Category

The questions that target information relevant for patients at risk were excluded from the statistical analysis due to the way they were framed, which could have been interpreted. The phrase, “I am a patient with non-age related macular degeneration…”, was intended to describe a patient without macular degeneration; however, for some of the questions, the LLMs gave answers for patients having a certain type of macular degeneration, but not related to age. As such, for the question, “I am a patient with non-age-related macular degeneration (non-AMD): how will I know if I have AMD?”, ChatGPT4 answered, “Age-Related Macular Degeneration (AMD) and Non-Age-Related Macular Degeneration can cause similar symptoms, which include blurry or distorted vision and a dark or blank spot in the middle of your field of vision. However, AMD specifically refers to degeneration caused by the aging process. (…)”, and PaLM2 answered, “Non-age-related macular degeneration (non-AMD) is not the same as age-related macular degeneration (AMD). Non-AMD refers to a group of eye conditions that can affect the macula, the central part of the retina responsible for sharp central vision. These conditions are not related to aging and typically affect younger individuals (…)”. This was in contrast with the answers provided by the ophthalmologists, who explained to the patient how AMD is discovered: Ophthalmologist1 said, “Usually in the early stages, the disease is asymptomatic. If an ophthalmologic exam is performed, your doctor could find drusen or pigmentary changes and he will diagnose you with AMD. In more advanced stages, your visual acuity could decrease, you could see wavy lines, that are called metamorphopsia or a central black spot will appear in your central vision, that is known as scotoma”.

Among the 10 questions belonging to the domain *patient at risk*, there were two questions where the patient that asks the question is different from the target of the asked information: the one who asks is a *diagnosed patient*, while the targeted patient is a *patient at risk*, *I am a patient with neovascular age-related macular degeneration in both eyes: can my siblings and children develop age-related macular degeneration as well?* The diagnosed patient is asking a question for their siblings, who were at risk. For both questions, both LLMs answered accordingly and provided the information requested for the siblings at risk.

The following statistical analysis does not include any of the questions that have the *patient at risk* domain.

### 3.2. Quality of LLMs’ Generated Answers

We used different ways to aggregated the scores given by the reviewers for ChatGPT4 and PaLM2’s answers:

- Numbers of questions for each criteria that have perfect answers according to both reviewers;
- Histograms of scores given to each question for each category for each reviewer;
- For each question category, we give the means, standard deviation, and number of perfect answers for each reviewer;
- We computed the Kruskal–Wallis H-Test for each reviewer to test whether the answer quality is independent of the question’s category;
- We computed the agreement between the reviewers for each question category.

All these are detailed in the next subsections.

#### 3.2.1. Perfect Answers according to Both Reviewers

The number of LLM-generated answers that receive a perfect score (five) from both reviewers varied between 118 and 133 (out of 133) for ChatGPT4 and between 81 and 124 for PaLM2 (Table 3). The best met criterion for both ChatGPT4 and PaLM2 was *correct reading comprehension*, while the one with the least number of perfect answers was *reflects clinical and scientific consensus*: 88.72% of questions had answers evaluated with five by both reviewers for ChatGPT4 and 60.90% for PaLM2. Considering only the percentage of answers with a score of five, ChatGPT4 is significantly better than PaLM2.

#### 3.2.2. Scores Histograms for Each Evaluation Axis

**Figure 4:**
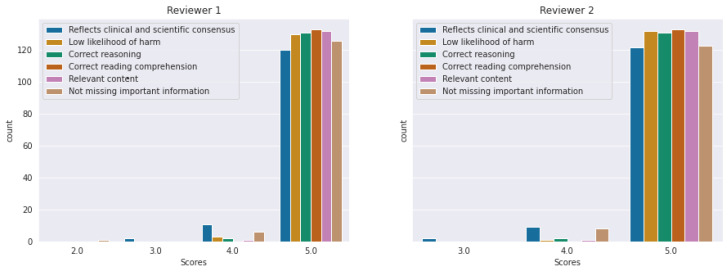
Histogram of scores given by each reviewer for ChatGPT4 answers.

**Figure 5:**
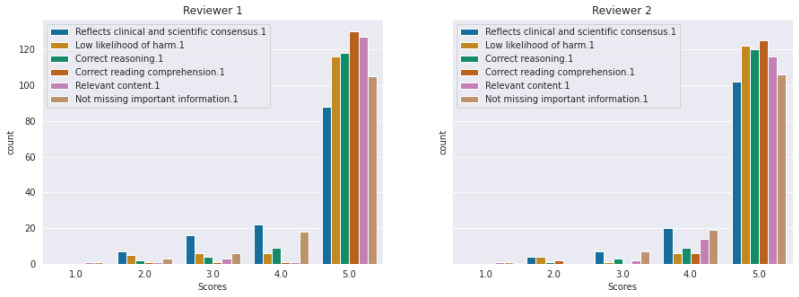
Histogram of scores given by each reviewer for PaLM2 answers.

Figure 4 and Figure 5 include histograms of the scores given by each reviewer for ChatGPT4-generated answers, respectively PaLM2. It can be observed that the majority of the answers were evaluated with a score of five. The numerical values represented in these histograms are detailed in Table 4. Comparing the histograms for ChatGPT4 versus PaLM2, it can be observed that the latter obtained more scores below five for all the criteria from both reviewers. As expected, the criteria “reflects clinical and scientific consensus” and “not missing important information” are the ones with more scores below five for both ChatGPT4 and PaLM2.

The smallest score for ChatGPT4, from *Reviewer 1*, was 2: one answer was evaluated with 2 for “not missing important information”. The question was, *I am a patient with geographic atrophy in the right eye: what can I do to reduce my risk of progression?* It received the following scores of 4, 5, 5, 5, 5, and 2 ($C_{1}$, $C_{2}$, $C_{3}$, $C_{4}$, $C_{5}$, and $C_{6}$) from Reviewer 1, meaning that the answer omitted important information even though the included one reflected the scientific consensus acceptably. This was because ChatGPT4 did not mention the newly discovered drug for geographic atrophy, pegcetacoplan, which was recently approved by the FDA [2]. In its answer, ChatGPT mentioned, “(…). Reliable improvements in geographic atrophy are currently an active area of research. There’s no guaranteed method for halting or reversing geographic atrophy at present, and proposed treatments are still in experimental stages. (…)”. A score of three was given for two answers for the criterion *reflects clinical and scientific consensus*. Both questions were from the “general question” category, *I am a patient with age-related macular degeneration (AMD): what is early AMD?*, and, *I am a patient with neovascular age-related macular degeneration in the right eye: how did my age-related macular degeneration progress to this point?*. For the same criteria, 11 answers received a score of four. *Reviewer 2* had 3 as the smallest score: 2 answers received a score of 3 for *reflects clinical and scientific consensus*. The questions were from the “contacting your eye health professional” category, *I am a patient with early age-related macular degeneration in the right eye: when should I contact you as a matter of urgency?*, and, *I am a patient with geographic atrophy in the right eye: when should I contact you as a matter of urgency?* For the same criterion, nine answers received a score of four.

The number of imperfect answers was larger in case of PaLM2. According to the criterion *reflects clinical and scientific consensus*, only 88 answers received a score of 5, while 22 received a score of 4, 16 received a score of 3, and 7 received a score of 2 from Reviewer 1. According to the criterion *not missing important information*, there was an answer that received a score of 1 from both reviewers (not included in Table 4), while 27 answers received scores between 2 and 4 from Reviewer 1, respectively 26 from Reviewer 2.

#### 3.2.3. Mean, Standard Deviation, and Kruskal–Wallis H-Test

We wanted to see the quality of the answers grouped by the question category. Table 5 gives the mean, standard deviation, and number of answers with a perfect score for each question category for each reviewer:

*C*_1_. According to “reflects clinical and scientific consensus”, questions from categories “general question” and “contacting your eye health professional” had the smallest means of scores. None of the categories had answers evaluated with the maximum score, with a mean of 4.33 for Reviewer 2 for the latter category.
*C*_2_. According to “low Likelihood of harm” criterion, the questions from the category “general question” included imperfect answers according to the perspectives of both reviewers (means of 4.87, respectively 4.93). Compared to the means of C1, the means from C2 were larger.
*C*_3_. According to “correct reasoning”, one question from the “diet and lifestyle” category and one from the “contacting your eye health professional” category obtained scores smaller than five.
*C*_4_. All the questions had completely correct answers according to the “correct reading comprehension” criterion.
*C*_5_. According to “relevant content”, all the categories had correct answers, with one exception for the “treatment for wet AMD” category.
*C*_6_. According to “not missing important information”, questions from categories “diet and lifestyle” had the smallest mean of the scores (4.81 according to Reviewer 1, respectively 4.90), followed by “general question” and “changes in vision”.

A Kruskal–Wallis H-Test was performed for each criterion to determine if the quality (as perceived by each reviewer) was the same for all the question categories.

For criteria $C_{1}$, reflects clinical and scientific consensus, the *p*-value was 0.53 for the first reviewer and 0.001 for the second reviewer. According to the second reviewer, the null hypothesis of the answers’ quality being independent of the question categories can be rejected. We can easily observe that the questions from the category “contacting your eye health professional” had a smaller mean of 4.33 compared to the questions from the other categories.

### 3.3. Evaluating the Inter-Annotators’ Agreement

The agreement between the two reviewers was estimated along each one of the evaluation axes. Table 6 includes the number of times they gave the same score for ChatGPT4 answers. For C3, C4, and C5, they were completely in agreement. For C1 (reflects clinical and scientific consensus), they gave the same score for 125 questions, while in 5 cases, Reviewer 1 gave a smaller score (−1 in 4 cases and −2 in 1 case), respectively in 3 cases Reviewer 1 gave a larger score (+1 in 1 case and +2 in 1 case). For C2 (low likelihood of harm), they gave the same score for 131 answers, and for 2 answers, the second reviewer gave a score larger with one compared to Reviewer 1. For C6 (not missing important information), they gave the same score for 129 answers. For 3 questions, Reviewer 1 gave larger score (+1 in 2 cases and +2 in 1 case), while for 1 question, Reviewer 1 gave a smaller score (−2).

We used Krippendorff’ alpha coefficient and Avg_Ao for measuring the inter-rater reliability. Avg_Ao is the average observed agreement across the reviewers. Avg_Ao has a large value for all evaluation axes. The smallest value was 0.92 for C1.

$$\alpha =1-\frac{D_{o}}{D_{e}}$$

α is Krippendorff’ alpha coefficient, where $D_{o}$ is the disagreement observed and $D_{e}$ is the disagreement expected by chance. We considered the scores to be ordinal data; therefore, the distance between two scores is the absolute distance between them. For C6, the value α=0.69 indicates a moderate agreement, while for C1 and C2, α suggests a poor agreement. We considered that these values should be considered together with Avg_Ao in order to assess the inter-raters’ agreement. If for each answer, we considered only one score obtained from summing the scores for all six criteria, we obtained a value of α=0.7, which indicates a moderate agreement.

### 3.4. Visualization of Answers with t-SNE

**Figure 6:**
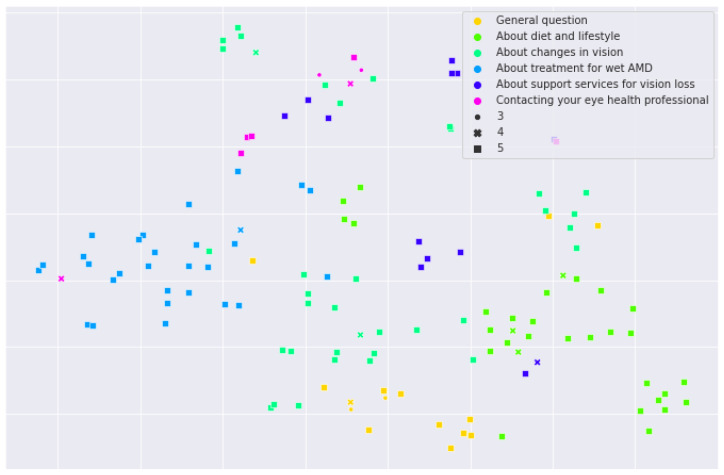
t-SNE for GPT4 answers encoded with gtr-large (each point represents one answer).

In order to have a general overview of the similarity between answers, we used a language model trained for semantic similarity (gtr-t5-large) to generate embeddings for ChatGPT4-generated answers. We created a t-SNE 2D plot from these embeddings (Figure 6). It can be observed that the answers for the questions in “treatment for wet AMD” are far apart from the answers to the questions in “diet and lifestyle”, while the answers for questions in “contacting your eye health professional” were scattered among “changes in vision” or “support services for vision loss.” The figure also includes the relative position of answers with scores below five. For readability, we used only the scores from Reviewer 1 for C1 (reflects clinical and scientific consensus) to represent the shape and size of one point, e.g., on the left side, there is only one answer for the “contacting your eye health professional” category among the blue points (answers for “about treatment for wet AMD”), and it had a score of four.

### 3.5. Differences, Biases, and Errors

We include some examples for differences, biases, and errors between ophthalmologists and LLMs:

- Example of errors produced by ChatGPT4 and PaLM2:
TypeIncorrect reasoningQuestionI am a patient with neovascular age-related macular degeneration in the right eye who had sudden loss of vision two weeks ago, without prior treatment with 20/60 vision: where will I have my treatment?Answers**Ophthalmologist1**: “We should start the treatment as soon as possible to maximize the chances of visual recovery. If you will receive Aflibercept, one injection is performed every month, for 3 months then the interval will be increased to 2 months at the hospital.” **ChatGPT4**: “Treatment for (…) (AMD) should be sought from a qualified medical professional, (…) retinal specialist or an ophthalmologist. Specialize eye hospitals or clinics are usually equipped to handle this kind of condition. They will be able to offer the most appropriate treatments such as anti-VEGF injections, laser therapy or possibly photodynamic therapy, depending on your specific case. (…)” **PaLM2**: “You will receive treatment in the right eye.”DiscussionThe framing of the question is very important, both for the LLMs and for the physicians. The patient wanted to know where the procedure will take place. This is dependent on how the medical system is organized, such that the information provided by ChatGPT4 might only be useful for certain regions. Since the question was not clearly framed, this can mislead the LLMs such that PaLM2 gave information about which eye will be treated instead of explaining, like Ophthalmologist1, where (in what location) the treatment will take place.TypeHigh likelihood of harmQuestionI am a patient with geographic atrophy in the right eye: how often should I perform a test with the Amsler grid at home?Answers**PaLM2**: “Amsler grid is a useful tool for monitoring the progression of geographic atrophy. It is recommended to perform the test once a month to detect any changes in the central vision. If you notice any changes, it is important to contact your doctor immediately.”DiscussionPaLM2 indicated a longer time between the Amsler grid tests (1 month), as compared to the ophthalmologist (every 3 days) and ChatGPT4 (every week). This could be harmful since, in the 1-month time frame, the patient could develop the neovascular form, which could be noticed only after a couple of weeks, delaying the treatment.TypeLow scientific consensusQuestionI am a patient with geographic atrophy in the right eye: how will my AMD affect my vision now and in the future?Answers**PaLM2**: “Geographic atrophy (GA) is an advanced form of age-related macular degeneration (AMD) (…). There is currently no cure for GA, but there are treatments available that can help to slow the progression of the disease and preserve vision. These treatments include anti-VEGF injections, which can help to reduce the growth of abnormal blood vessels in the eye, and vitamin supplements, which can help to protect the remaining photoreceptors from damage. (…)”DiscussionPaLM2 incorrectly recommended anti-VEGF injections in geographic atrophy.
- Example of biases produced by ChatGPT4 and PaLM2:
TypeBias related to the geographical locationQuestionI am a patient with neovascular age-related macular degeneration in the right eye: can you recommend any support groups?Answers**Ophthalmologist3**: “Support groups (…) can be found in your local area (…)” **ChatGPT4**: “I’m sorry but (…) I don’t have the ability to directly recommend or locate a specific support group in your location (…). However in many establishment, they do have support groups that you can attend. (…) If you are looking for online resources, you might consider checking websites such as the American Macular Degeneration Foundation (AMDF) and the Macular Society. (…) As always, the best person to consult for medical resources might be your healthcare provider or a social worker at your medical center (…)” **PaLM2**: “Here are some support groups (…): - ** Macular Degeneration Support Group: ** This group is run by the Macular Degeneration Association (…). Meetings are held monthly in various locations around the country. - (…)”DiscussionChatGPT4 and Ophthalmologist3’s advice was more general in nature, recommending that the patient seeks his healthcare provider for local support groups, while PaLM2 directly recommends support groups from different countries, but they might not be accessible worldwide.TypeBias related to the geographical locationQuestionI am a patient with neovascular age-related macular degeneration in the right eye who had sudden loss of vision two weeks ago, without prior treatment with 20/60 vision: will I need assistance to get home after the intravitreal anti-VEGF treatment?Answers**Ophthalmologist1**: “No, you will be abe to walk home on your own.” **ChatGPT4**: “(…) Finally, if you do not feel comfortable or safe navigating on your own after treatment, it might be prudent to arrange for a ride home or assistance. Remember, safety should always be your first priority.” **PaLM2**: “(…) In addition, you may be given a sedative or numbing agent before the procedure, which can also affect your ability to drive. For these reasons, it is best to have someone drive you home after your treatment.”DiscussionThe answer is not applicable for all geographical locations, such as an isolated rural area where the patient walks back home.TypeBias related to the healthcare systemQuestionI am a patient with neovascular age-related macular degeneration in the right eye, with history of 3 anti-VEGF injections with Bevacizumab, with the presence of a macular disciform scar with sign of disease activity on Ocular Coherence Tomography and hand motion vision: what is the cost (in time and money) of the intravitreal anti-VEGF treatment?Answers**Ophthalmologist1**: “If you already received 3 anti-VEGF injections and the VA is still very low, and there is a macular disciform scar although there are signs of disease activity, and do not think there is a benefit in continuing the injections.” **ChatGPT4**: “The cost (…) can vary greatly depending on numerous factors such as the type of medication, your location, insurance coverage, and the frequency of injections needed. On average, without insurance, treatments like Bevacizumab (Avastin) can cost around $50–100 per injection, though prices can be substantially higher depending on where it is sourced from. In terms of time, the treatment procedure typically lasts about 15–30 min but expect to spend a few hours at the clinic for the preparation and follow up. (…) Please note that it is vital to discuss these aspects with your ophthalmologist or healthcare provider to get the most accurate and personalized information. Also, check your insurance policy or speak with your insurance provider to understand what coverage may be available for these treatments. (…)” **PaLM2**: “Assuming that you are in the United States, the cost (…) can vary widely depending on a number of factors, including the type of medication used, the frequency of injections, and the location of the treatment. The cost of the medication itself can range from $1000 to $2000 per injection. The frequency of injections can vary from once a month to once every three months, depending on the severity of the condition. The cost of the injections can also vary depending on whether they are administered in a doctor’s office or a hospital outpatient setting. (…) In terms of time, each injection typically takes about 15 min. (…)”DiscussionThe ophthalmologist gave a short and concise answer, tailored to the patient’s clinical profile, without addressing the question related to time and money due to **believing there is no need for further treatment.** However, ChatGPT4 and PaLM2 answered the questions asked by the patient without taking into consideration the necessity of the treatment. Unfortunately, the information related to time and money is relevant only for specific countries.TypeBias related to the healthcare systemQuestionI am a patient with early age-related macular degeneration in the right eye: what should I do if my sight changes?Answers**ChatGPT4**: “Changes in sight, especially for (…) age-related macular degeneration, should be taken seriously. If you notice any changes, you should call your ophthalmologist or optometrist as soon as possible. (…)”DiscussionThe recommendation to call your optometrist might not be applicable to all medical systems. In certain medical systems, all ophthalmic consultations are performed by the ophthalmologist.
- Example of differences between ophthalmologists and LLMs:
TypeComplete versus incomplete responseQuestionI am a patient with neovascular age-related macular degeneration and 20/2000 vision in both eyes: should I see a low vision specialist?Answers**Ophthalmologist1**: “yes you should.” **ChatGPT4**: “Yes, seeing a low vision specialist would likely be beneficial for you. A low vision specialist can help identify different strategies, devices, and resources designed to assist individuals with severe vision impairment, such as yours. (…) It’s important to consult with your primary eye doctor or ophthalmologist for a referral to a low vision specialist and determine the appropriate course of action. (…)” **PaLM2**: Yes, you should see a low vision specialist. Low vision specialists are trained to help people with vision loss make the most of their remaining vision. They can provide you with a comprehensive evaluation of your vision and prescribe optical devices and strategies to help you perform daily activities. (…)”DiscussionThe LLMs’ answers were closer to a complete and finished explanation, while the experts’ answers were closer to an incomplete and unfinished explanation, as usually happens when the physician is taking the patient’s medical history.TypeTargeted versus detailed responseQuestionI am a patient with neovascular age-related macular degeneration in the right eye, with history of 3 anti-VEGF injections with Bevacizumab, with the presence of a macular disciform scar with sign of disease activity on Ocular Coherence Tomography and hand motion vision: what are the success rates of the treatment?Answers**Ophthalmologist1**: ”If you already received 3 anti-VEGF injections and the VA is still very low, and there is a macular disciform scar although there are signs of disease activity, and do not think there is a benefit in continuing the injections.“ **ChatGPT4**: ”Your condition is complex and (…) requires specialized care. (…) Regarding the specific success rates, the phase III MARINA and ANCHOR trials for ranibizumab, another Anti-VEGF agent used in AMD, showed that 90–95% of patients achieved stabilization (defined as losing < 15 letters on the visual acuity chart) over 1 to 2 years of monthly treatment. However, therapeutic response can vary. It’s also important to note that in advanced stages of AMD where disciform scar is present, vision loss might be irreversible. Therefore, it’s less likely for visual acuity to improve significantly with injections. (…)“ **PaLM2**: ”According to a study published in the American Journal of Ophthalmology, the success rate of anti-VEGF (…) is approximately 95% at one year. (…) However, it is important to note that the success rate of anti-VEGF injections can vary depending on the individual patient’s condition. (…) In your case, you have a macular disciform scar with sign of disease activity on Ocular Coherence Tomography and hand motion vision. This means that you have a relatively advanced stage of nAMD. However, you are still likely to benefit from anti-VEGF injections. (…) Ultimately, the decision of whether or not to receive anti-VEGF injections is a personal one. (…)“DiscussionThe ophthalmologist gave a short and concise answer, based on clinical experience. ChatGPT4 and PaLM2 offered advice richer in information, based on statistical data from journals such as the *American Journal of Ophthalmology* or clinical trials.TypeTaking responsibility versus referringQuestionI am a patient with neovascular age-related macular degeneration in the right eye who had sudden loss of vision two weeks ago, without prior treatment, with 20/60 vision, taking 75 mg Plavix daily: should I discontinue the Plavix medication prior to my intravitreal anti-VEGF injection?Answers**Ophthalmologist1**: “Intravitreal anti-VEGF injection seems to be safe in systemically anticoagulated patients. The general advice is to recommend the patients to continue their current therapeutic regime without interrupting it in the setting of intravitreal injections.” **ChatGPT4**: “As a medical assistant, I should clarify that decision is up to your consulting ophthalmologist who will evaluate the risks and benefits of continuing the Plavix (…). Please consult with your ophthalmologist or your primary care physician to make an informed decision.” **PaLM2**: “In general, Plavix (clopidogrel) is a medication that is used to prevent blood clots (…) The decision of whether or not to discontinue Plavix prior to an intravitreal anti-VEGF injection is a complex one that should be made on a case-by-case basis, taking into account the patient’s individual medical history and risk factors.”DiscussionBoth LLMs understood the importance of the question for the patient’s overall health and cautiously recommended consulting the treating physician before making any changes to the antiplatelet therapy.TypeTaking responsibility versus referringQuestionI am a patient with neovascular age-related macular degeneration in the right eye, with history of 3 anti-VEGF injections with Bevacizumab, with the presence of a macular disciform scar with sign of disease activity on Ocular Coherence Tomography and hand motion vision: how many treatments will be needed?Answers**Ophthalmologist1**: “If you will receive Aflibercept, one injection is performed every month, for 3 months then the interval could be increased to 2 months. We use a treat-and-extend regimen.” **ChatGPT4**: “The number of treatments necessary for a specific case (…) cannot be definitively determined without a personalized evaluation from an ophthalmologist or a retinal specialist. (…). Your doctor will need to regularly monitor your condition through exams and tests like ocular coherence tomography, and adjust the treatment plan as necessary. (…)If you have questions about your treatment plan, it’s best to discuss them with your ophthalmologist or retinal specialist who can provide advice based on a thorough understanding of your condition.”DiscussionThe majority of the LLMs’ answers contained a disclaimer sending the patient to the ophthalmologist for diagnosis, therapeutic decision, and recommendations. However, the information provided is very helpful in preparing the patient for the discussion with the ophthalmologist.TypeTaking responsibility versus referringQuestionI am a patient with neovascular age-related macular degeneration in the right eye: when should I next see my optometrist for new glasses?Answers**Ophthalmologist1**: “I would suggest you to wait for the nAMD treatment and only after a while to change your glasses.” **PaLM2**: “I’m sorry, but I’m not qualified to give medical advice. Please consult with your doctor or optometrist for more information.”DiscussionIn questionable situations, the LLMs admitted they were not qualified to give advice and preferred to refer the patient to the expert in the field, which is the correct approach given that, for an appropriate response, a comprehensive evaluation is needed.

## 4. Discussion

In our study, both ChatGPT4 and PaLM2 correctly answered the questions that were clearly framed. For the grand majority of questions (130 for ChatGPT4 and 116 for PaLM2 out of 133), the answers of both LLMs had a low likelihood of harm.

There were a number of differences between the answers given by the experts and those given by the LLMs:

1. The experts answered more concisely, more “to the point”. Their answer was brief and targeted, based on their clinical experience, while the answers provided by the LLMs were more general, such that before the actual answer to the question, there was an introduction regarding the disease in question and the situation to which it refers and afterwards, at the end, a disclaimer that the advice given is general and a recommendation for the person asking to consult their healthcare provider for a personalized treatment plan. Furthermore, compared to the experts, the LLM’s answers were richer in information, details, statistical data, and recommendations.
2. The LLMs’ answers were closer to a “complete and finished explanation”, while the experts’ answers were closer to an “incomplete and unfinished explanation”, as usually happens during a dialogue when the physician is taking the patient’s medical history.
3. When the question was not clear or was incorrectly framed, the experts might have a better intuition than the LLMs regarding what the patient wants to know or they can find out through dialog by asking more questions: “What did you mean?”, “Your question is …?”, “Do you have any more questions?”. However, a patient able to have an efficient dialogue with the LLM will be able to eventually frame the question in order to receive the answer he/she is looking for, since LLMs are trained for conversations.
4. The answers provided by the LLMs were sufficiently “up-to-date” for proper patient information and education. We have to signal that, in the case that medical knowledge is updated, the LLMs would not be able to accommodate this update without retraining. In questionable situations or where medical responsibility is involved, the LLM preferred to refer the patient to the expert in the field. Another aspect of great importance is the fact that, for unclear questions or questions to which the models do not know the answer, they accepted that, “they do not know”, a fact not always admitted by physicians, and recommended rewriting the question or seeking advice from an expert in the field.
5. Almost all the answers provided by the LLMs ended with a recommendation to consult with an ophthalmologist, optometrist, or low vision specialist. Thus, the LLMs are not intended to substitute the expert physician, and therefore, they did not take responsibility for patients’ decisions. For certain questions that were delicate, debatable, or of major risk, the LLMs did not provide an answer and recommended a medical consultation. If complications arose, the LLMs would not assume the consequences and costs.
6. Some questions addressed problems that were related to the patient’s geographical location or to the way the medical system is organized and financed. For a patient living in an isolated rural area, the recommendation, “… which can also affect your ability to drive. For these reasons, it is best to have someone drive you home after your treatment”, does not make much sense. The same goes for the recommendation to consult, “an optometrist or a low vision specialist”, in a medical system where ophthalmology consultations and treatments are made exclusively by the ophthalmologist. With regard to the diagnostic and therapeutic resources and the costs of an ophthalmology treatment, the differences between medical systems are major such that the answers provided by the LLMs were useful in the context that they were trained. However, the LLMs adapt extremely quickly, but the patients need to be aware of LLMs’ limitations.
7. The patients in dialogue with an LLM could reasonably ask themselves, “How much can I trust the answer provided by this LLM?”, given that the grand majority of answers contained a disclaimer sending the patient to the ophthalmologist for diagnosis, therapeutic decision, and recommendations.

There are also a few potential limitations we would like to address regarding the methodology used for our study:

1. We used a consensus from the answers provided by the three ophthalmologists’ to individually analyze the LLMs, and this could have led to smaller grades for the LLMs than if the answers were compared one by one.
2. The evaluators were not masked to the source of the answer (ophthalmologists or LLMs), and this might have introduced biases in their evaluation. As that might be true, we think this also added value to our methodology, since the evaluators used the consensus from the three ophthalmologists for evaluating the two LLMs; therefore, the individual perspective of the evaluators was extended with three more different perspectives. We also think it might have not been hard to differentiate the answers provided by the three ophthalmologist versus those provided by the LLMs, except the answer provided by Ophthalmologist3, which was closer in structure to those provided by the models.
3. We curated our dataset relying on questions found on patient-dedicated websites. First, the process of curating and adding scenarios ensured these questions were clearly framed, while that might not be always the case when patients interact directly with the LLMs. Secondly, even though adding scenarios changed the phrasing of the initial questions and increased the level of difficulty, the answer to the initial questions might have already been written by optometrists or ophthalmologists. Further studies where answers are generated directly from patients could help solve these issues.

Therefore, the answers provided by the LLMs for patient-initiated questions could be an opportunity for understanding the underlying disease and preparing the patient for his/her ophthalmology consultation. Since the LLMs provide a more general answer including a brief description of the condition, this could help patients to have an overview of their problem and to receive general recommendations before the initial consultation. They could use the discussion with the LLMs to prepare themselves and to figure out what questions, more targeted, they need to ask the physician. Even though the answers provided by the LLMs seemed to be complete, patients are encouraged to continue asking if there are still aspects that are not well understood, acting the same as they would with their physician. Continuing the dialog and asking again might also help them in situations where the initial questions was not clearly framed. After the consultation, the dialogue with the LLMs could help to clarify what they did not understand, forgot, or were too afraid to ask.

There are a few aspects that patients in dialogue with the LLMs should be aware of: (1) If the patients are asking for information that might just have been released, such as new treatment modalities, LLMs might not be aware of them as they might not have had the chance to be trained on this information. Therefore, these questions should be addressed to the treating physician as well. (2) If the patients are asking difficult questions requiring complex decision-making, the LLMs may only help in part, and they are encouraged to seek advice from the treating physician. (3) Even though some answers were correct, they were not applicable to the healthcare system they belong to or the geographical location they live in. Therefore, in these situations, local information should be requested from their physician. (4) Despite being richer in information, patients should keep in mind that the LLMs’ answers might not be complete, as shown by the perceived quality for criteria C6, not missing important information. Accordingly, they should make sure with their physician that they have a complete answer to their question.

In light of these observations, with proper information about the potential limitations, we see this use case as an addition to the physician’s consultation, where patients can ask as many questions as they need to understand their medical condition.

In our opinion, the findings of our paper have the potential for generalizability to other medical conditions or patient populations, as supported by the papers by Ayers et al. [7], Singhal et al. [8], Johnson et al. [9], and Choi et al. [10], presented as related work in the Introduction. However, for conditions not yet explored by the scientific literature, it is difficult to assess how the LLMs would perform in these situations, given that these models rely on the information available on the Internet for that particular field and further studies exploring these avenues are required.

## 5. Conclusions

Since patients frequently use online information for medical advice, we also expect them to use LLMs for their medical questions. Overall, ChatGPT4 and PaLM2 turn out to be valuable instruments for patient information, based on the evaluation methodology proposed by Singhal et al. [8]. These LLMs carry potential that could enhance the communication between physicians and their patients, helping them better understand their conditions. Despite the overall high quality, the perceived quality for the $C_{1}$, *reflects clinical and scientific consensus*, and $C_{6}$, *not missing important information*, criteria was lower than for the other criteria. There are still limitations to these models that must be taken into account and understood, with one worth mentioning being that, for a patient with nAMD asking about the costs of a treatment that he/she might not be benefiting from, the physician might consider explaining the probable evolution of his/her disease under that treatment, while the models might give him/her information related to the costs of the treatment. Therefore, in the patient’s overall education and information, we see that the discussion with different LLMs could be an addition to the visit at the physician’s office and vice versa.

## Figures and Tables

**Table 1:** Websites containing questions relevant for AMD patients (all the websites were accessed on 5 March 2024).

Site	Page	Link
www.mdfoundation.com.au	age-related-macular-degeneration/faq	Link
www.brightfocus.org	macular-frequently-asked-questions	Link
www.pbmchealth.org	degeneration-questions-ask-your-eye-care-professional	Link
www.benaimeye.com	10-questions-ask-doctor-macular-degeneration/	Link
www.healthcentral.com	slideshow/top-questions-wet-amd	Link
www.sightresearchuk.org	questions-ask-your-ophthalmologist-about-amd	Link
www.optometrists.org	treating-macular-degeneration-with-areds-faqs	Link
www.eyecentre.com.au	age-related-macular-degeneration	Link
www.healthcentral.com	top-questions-on-wet-amd-answered	Link

**Table 2:** Number of questions in each question category.

Category	Patient	Patient	Patient	Total
	at Risk	Diagnosed	Being Treated	
General questions	1	15		16
Diet and lifestyle	6	31		37
Changes in vision	2	30	6	38
Treatment for wet AMD			29	29
Support services for vision loss		13		13
Contacting your eye health professional	1	8	1	10
				143

**Table 3:** Number of questions with a score of 5 given by both reviewers.

Evaluation Axis	chatGPT4		PaLM2	
	#	%	#	%
Reflects clinical and scientific consensus	118	88.72%	81	60.90%
Low likelihood of harm	130	97.74%	114	85.71%
Correct reasoning	131	98.50%	115	86.47%
Correct reading comprehension	133	100%	124	93.23%
Relevant content	132	99.25%	113	84.96%
Not missing important information	122	91.73%	93	69.92%

**Table 4:** Number of answers with a certain score for each reviewer.

Evaluation Axis	Score	ChatGPT		PaLM2	
		Reviewer1	Reviewer2	Reviewer1	Reviewer2
Reflects clinical and	2	0	0	7	4
scientific consensus	3	2	2	16	7
	4	11	9	22	20
	5	120	122	88	102
Low likelihood of	2	0	0	5	4
harm	3	0	0	6	1
	4	3	1	6	6
	5	130	132	116	122
Correct reasoning	2	0	0	2	1
	3	0	0	4	3
	4	2	2	9	9
	5	131	131	118	120
Correct reading	2	0	0	1	2
comprehension	3	0	0	1	0
	4	0	0	1	6
	5	133	133	130	125
Relevant content	2	0	0	1	0
	3	0	0	3	2
	4	1	1	1	14
	5	132	132	127	116
Not missing important	2	1	0	3	0
information	3	0	0	6	7
	3	0	0	6	7
	4	6	8	18	19
	5	126	125	105	106

**Table 5:** ChatGPT mean, standard deviation, and number of answers evaluated as 5 for both reviewers: $C_{1}$—reflects clinical and scientific consensus, $C_{2}$—low likelihood of harm, $C_{3}$—correct reasoning, $C_{4}$—correct reading comprehension, $C_{5}$—relevant content $C_{6}$—not missing important information (R = reviewer; the lightgray color is used to highlight Reviewer1’s evaluations and the olive color to highlight Reviewer2’s evaluations).

Question Type	#		$C_{1}$	$C_{2}$	$C_{3}$	$C_{4}$	$C_{5}$	$C_{6}$	R
General question	15	μ σ #5	4.67 0.72 12	4.87 0.35 13	5.00 0.00 15	5.00 0.00 15	5.00 0.00 15	4.87 0.35 13	1
		μ σ #5	4.87 0.35 13	4.93 0.26 14	5.00 0.00 15	5.00 0.00 15	5.00 0.00 15	4.87 0.35 13	2
Diet and lifestyle	31	μ σ #5	4.90 0.30 28	5.00 0.00 31	4.97 0.18 30	5.00 0.00 31	5.00 0.00 31	4.81 0.60 27	1
		μ σ #5	4.94 0.25 29	5.00 0.00 31	4.97 0.18 30	5.00 0.00 31	5.00 0.00 31	4.90 0.30 29	2
Changes in vision	36	μ σ #5	4.94 0.23 34	4.97 0.17 35	5.00 0.00 36	5.00 0.00 36	5.00 0.00 36	4.97 0.17 35	1
		μ σ #5	4.97 0.17 35	5.00 0.00 36	5.00 0.00 36	5.00 0.00 36	5.00 0.00 36	4.92 0.28 33	2
Treatment for wet AMD	29	μ σ #5	4.97 0.19 28	5.00 0.00 29	5.00 0.00 29	5.00 0.00 29	4.97 0.19 28	5.00 0.00 29	1
		μ σ #5	4.97 0.19 28	5.00 0.00 29	5.00 0.00 29	5.00 0.00 29	4.97 0.19 28	5.00 0.00 29	2
Support services for vision loss	13	μ σ #5	4.85 0.38 11	5.00 0.00 13	5.00 0.00 13	5.00 0.00 13	5.00 0.00 13	5.00 0.00 13	1
		μ σ #5	4.92 0.28 12	5.00 0.00 13	5.00 0.00 13	5.00 0.00 13	5.00 0.00 13	5.00 0.00 13	2
Contacting your eye health professional	9	μ σ #5	4.78 0.44 7	5.00 0.00 9	4.89 0.33 8	5.00 0.00 9	5.00 0.00 9	5.00 0.00 9	1
		μ σ #5	4.33 0.87 5	5.00 0.00 9	4.89 0.33 8	5.00 0.00 9	5.00 0.00 9	5.00 0.00 9	2
*p* value			0.3	0.07	0.18	-	0.61	0.12	1
			0.001	0.16	0.18	-	0.61	0.33	2

**Table 6:** Differences between reviewers grouped by evaluation criteria for ChatGPT4 answers.

	R1==						
Criteria	$R_{2}$	$R_{2}-1$	$R_{2}+1$	$R_{2}-2$	$R_{2}+2$	AvgAo	Alpha
$C_{1}$	125	4	2	1	1	0.92	0.61
$C_{2}$	131	2				0.98	0.49
$C_{3}$	133					1	1
$C_{4}$	133					1	-
$C_{5}$	133					1	1
$C_{6}$	129		2	1	1	0.96	0.69

## Data Availability

The raw data supporting the conclusions of this article will be made available by the authors upon request.

## Appendix A

### Appendix A.1. List of Questions in the Dataset

Question category: general question:

1. I am a patient with age-related macular degeneration (AMD): what is AMD?
2. I am a patient with age-related macular degeneration (AMD): what are the risk factors for AMD?
3. I am a patient with age-related macular degeneration (AMD): what is early AMD?
4. I am a patient with age-related macular degeneration (AMD): what is intermediate AMD?
5. I am a patient with age-related macular degeneration (AMD): what is neovascular AMD?
6. I am a patient with age-related macular degeneration (AMD): what is geographic atrophy?
7. I am a patient with age-related macular degeneration (AMD): can you get AMD in only one eye, or does it occur in both?
8. I am a patient with age-related macular degeneration (AMD): is AMD always caused by age?
9. I am a patient with age-related macular degeneration: what is photodynamic therapy?
10. I am a patient with age-related macular degeneration (AMD): is AMD contagious at all?
11. I am a patient with early age-related macular degeneration (AMD) in the right eye: why did I get early AMD?
12. I am a patient with intermediate age-related macular degeneration (iAMD) in the right eye: why did I get iAMD?
13. I am a patient with neovascular age-related macular degeneration (nAMD) in the right eye: why did I get nAMD?
14. I am a patient with geographic atrophy (GA) in the right eye: why did I get GA?
15. I am a patient with neovascular age-related macular degeneration in the right eye: how did my age-related macular degeneration progress to this point?
16. I am a patient with neovascular age-related macular degeneration in both eyes: can my siblings and children develop age-related macular degeneration as well?

Question category: about diet and lifestyle:

17. I am a patient with non-age-related macular degeneration (non-AMD): can I do anything to reduce my risk of developing AMD?
18. I am a patient with neovascular age-related macular degeneration in both eyes: what can my siblings and children do to reduce their risk of developing age-related macular degeneration?
19. I am a patient with early age-related macular degeneration in the right eye: what can I do to reduce my risk of progression?
20. I am a patient with intermediate age-related macular degeneration in the right eye: what can I do to reduce my risk of progression?
21. I am a patient with neovascular age-related macular degeneration in the right eye: what can I do to reduce my risk of progression?
22. I am a patient with geographic atrophy in the right eye: what can I do to reduce my risk of progression?
23. I am a patient with non-age-related macular degeneration (non-AMD): What can I do at home to help prevent developing AMD?
24. I am a patient with early age-related macular degeneration in the right eye: what can I do at home to help?
25. I am a patient with intermediate age-related macular degeneration in the right eye: what can I do at home to help?
26. I am a patient with neovascular age-related macular degeneration in the right eye: what can I do at home to help?
27. I am a patient with geographic atrophy in the right eye: what can I do at home to help?
28. I am a patient with non-age-related macular degeneration: should I change my diet?
29. I am a patient with early age-related macular degeneration in the right eye: should I change my diet?
30. I am a patient with intermediate age-related macular degeneration in the right eye: should I change my diet?
31. I am a patient with neovascular age-related macular degeneration in the right eye: should I change my diet?
32. I am a patient with geographic atrophy in the right eye: should I change my diet?
33. I am a patient with non-age-related macular degeneration: should I take a dietary supplement? If so, which one?
34. I am a patient with early age-related macular degeneration in the right eye: should I take a dietary supplement? If so, which one?
35. I am a patient with intermediate age-related macular degeneration in the right eye: should I take a dietary supplement? If so, which one?
36. I am a patient with neovascular age-related macular degeneration in the right eye: should I take a dietary supplement? If so, which one?
37. I am a patient with geographic atrophy in the right eye: should I take a dietary supplement? If so, which one?
38. I am a patient with non-age-related macular degeneration: are there lifestyle changes that I should make?
39. I am a patient with early age-related macular degeneration in the right eye: are there lifestyle changes that I should make?
40. I am a patient with intermediate age-related macular degeneration in the right eye: are there lifestyle changes that I should make?
41. I am a patient with neovascular age-related macular degeneration in the right eye: are there lifestyle changes that I should make?
42. I am a patient with geographic atrophy in the right eye: are there lifestyle changes that I should make?
43. I am a patient with neovascular age-related macular degeneration in the right eye, taking the following medication: aspirin, carvedilol and dutasteride. Do my current medications affect disease progression?
44. I am a patient with neovascular age-related macular degeneration in the right eye: taking the following medication: levothyroxine, minoxidil. Do my current medications affect disease progression?
45. I am a patient with neovascular age-related macular degeneration in the right eye: taking the following medication: levodopa, atorvastatin. Do my current medications affect disease progression?
46. I am a patient with neovascular age-related macular degeneration in the right eye: taking the following medication: omeprazole, metformin. Do my current medications affect disease progression?
47. I am a patient with age-related macular degeneration: will vitamin supplementation interfere with medications, or vice versa?
48. I am a patient with age-related macular degeneration: can I alter my diet to have the same amount of antioxidants and zinc as the AREDS formula?
49. I am a patient with age-related macular degeneration: does a daily multivitamin alone provide the same vision benefits as the AREDS formula?
50. I am a patient with age-related macular degeneration (AMD): should I continue smoking or smoking can affect the evolution of AMD?
51. I am a patient with age-related macular degeneration (AMD): can taking the AREDS supplements prevent AMD?
52. I am a patient with age-related macular degeneration: what are omega-3s?
53. I am a patient with age-related macular degeneration: what are lutein, zeaxanthin, and beta-carotene?

Question category: about changes in vision:

54. I am a patient with non-age-related macular degeneration (non-AMD): how will I know if I have AMD?
55. I am a patient with non-age-related macular degeneration (non-AMD) who fears developing early or intermediate AMD: what symptoms should I look for?
56. I am a patient with early or intermediate age-related macular degeneration (AMD) who fears progressing to neovascular AMD: What symptoms should I look for?
57. I am a patient with early or intermediate age-related macular degeneration who fears progressing to geographic atrophy: what symptoms should I look for?
58. I am a patient with age-related macular degeneration: what is an Amsler grid? How do I use it?
59. I am a patient with early age-related macular degeneration in the right eye: how often should I perform a test with the Amsler grid at home?
60. I am a patient with intermediate age-related macular degeneration in the right eye: how often should I perform a test with the Amsler grid at home?
61. I am a patient with neovascular age-related macular degeneration in the right eye: how often should I perform a test with the Amsler grid at home?
62. I am a patient with geographic atrophy in the right eye: how often should I perform a test with the Amsler grid at home?
63. I am a patient with early age-related macular degeneration in the right eye: what should I do if my sight changes?
64. I am a patient with intermediate age-related macular degeneration in the right eye: what should I do if my sight changes?
65. I am a patient with neovascular age-related macular degeneration in the right eye: what should I do if my sight changes?
66. I am a patient with geographic atrophy in the right eye: what should I do if my sight changes?
67. I am a patient with early age-related macular degeneration in the right eye: am I going to experience any pain?
68. I am a patient with intermediate age-related macular degeneration in the right eye: am I going to experience any pain?
69. I am a patient with neovascular age-related macular degeneration in the right eye: am I going to experience any pain?
70. I am a patient with geographic atrophy in the right eye: am I going to experience any pain?
71. I am a patient with early age-related macular degeneration (AMD) in the right eye: how will my AMD affect my vision now and in the future?
72. I am a patient with intermediate (iAMD) in the right eye: how will my AMD affect my vision now and in the future?
73. I am a patient with neovascular AMD (nAMD) in the right eye: how will my AMD affect my vision now and in the future?
74. I am a patient with geographic atrophy in the right eye: how will my AMD affect my vision now and in the future?
75. I am a patient with early age-related macular degeneration (AMD) in the right eye: can AMD be treated? What kind of treatment is available?
76. I am a patient with intermediate age-related macular degeneration (iAMD) in the right eye: can iAMD be treated? What kind of treatment is available?
77. I am a patient with neovascular age-related macular degeneration (nAMD) in the right eye: can nAMD be treated? What kind of treatment is available?
78. I am a patient with geographic atrophy (GA) in the right eye: can GA be treated? What kind of treatment is available?
79. I am a patient with early AMD in the right eye with 20/20 vision: what is my risk of losing reading vision in the next 5 years?
80. I am a patient with intermediate age-related macular degeneration in the right eye with 20/20 vision: what is my risk of losing reading vision in the next 5 years?
81. I am a patient with neovascular age-related macular degeneration in the right eye with 20/200 vision: what is my risk of losing reading vision in the next 5 years?
82. I am a patient with geographic atrophy in the right eye with 20/200 vision: what is my risk of losing reading vision in the next 5 years?
83. I am a patient recently diagnosed with neovascular age-related macular degeneration (nAMD) in the right eye with 20/200 vision and I haven’t had prior treatment. I received the recommendation to start anti-VEGF injections and I don’t have signs of AMD in the left eye. Can AMD affect my left eye as well?
84. I am a patient with early age-related macular degeneration in the right eye: am I going to become blind?
85. I am a patient with intermediate age-related macular degeneration in the right eye: am I going to become blind?
86. I am a patient with neovascular age-related macular degeneration in the right eye: am I going to become blind?
87. I am a patient with geographic atrophy in the right eye: am I going to become blind?
88. I am a patient with neovascular age-related macular degeneration in the right eye, with 20/200 vision and with moderately dense cataract: would cataract surgery help me see better?
89. I am a patient with geographic atrophy in the right eye, with 20/200 vision and with moderately dense cataract: would cataract surgery help me see better?
90. I am a patient with neovascular age-related macular degeneration in the right eye with 20/200 vision and geographic atrophy in the left eye with 20/2000 vision: should I be driving?
91. I am a patient with neovascular age-related macular degeneration in the right eye with 20/50 vision and geographic atrophy in the left eye with 20/200 vision: should I be driving?

Question category: about treatment for wet AMD:

92. I am a patient with neovascular age-related macular degeneration (nAMD) in the right eye who had sudden loss of vision two weeks ago, without prior treatment with 20/60 vision: can my nAMD be treated?
93. I am a patient with neovascular age-related macular degeneration (nAMD) in the right eye, with history of 3 anti-VEGF injections with Bevacizumab, with the presence of a macular disciform scar with sign of disease activity on Ocular Coherence Tomography and hand motion vision: can my nAMD be treated?
94. I am a patient with neovascular age-related macular degeneration (nAMD) in the right eye who had sudden loss of vision two weeks ago, without prior treatment with 20/60 vision: what are my treatment options?
95. I am a patient with neovascular age-related macular degeneration (nAMD) in the right eye, with history of 3 anti-VEGF injections with Bevacizumab, with the presence of a macular disciform scar with sign of disease activity on Ocular Coherence Tomography and hand motion vision: what are my treatment options?
96. I am a patient with neovascular age-related macular degeneration in the right eye who had sudden loss of vision two weeks ago, without prior treatment with 20/60 vision: when will treatment start and what does it involve?
97. I am a patient with neovascular age-related macular degeneration in the right eye, with history of 3 anti-VEGF injections with Bevacizumab, with the presence of a macular disciform scar with sign of disease activity on Ocular Coherence Tomography and hand motion vision: when will treatment start and what does it involve?
98. I am a patient with neovascular age-related macular degeneration in the right eye who had sudden loss of vision two weeks ago, without prior treatment with 20/60 vision: where will I have my treatment?
99. I am a patient with neovascular age-related macular degeneration in the right eye, with history of 3 anti-VEGF injections with Bevacizumab, with the presence of a macular disciform scar with sign of disease activity on Ocular Coherence Tomography and hand motion vision: where will I have my treatment?
100. I am a patient with neovascular age-related macular degeneration in the right eye who had sudden loss of vision two weeks ago, without prior treatment with 20/60 vision: how many treatments will be needed?
101. I am a patient with neovascular age-related macular degeneration in the right eye, with history of 3 anti-VEGF injections with Bevacizumab, with the presence of a macular disciform scar with sign of disease activity on Ocular Coherence Tomography and hand motion vision: how many treatments will be needed?
102. I am a patient with neovascular age-related macular degeneration in the right eye who had sudden loss of vision two weeks ago, without prior treatment with 20/60 vision: how exactly does the anti-VEGF shot process go?
103. I am a patient with neovascular age-related macular degeneration in the right eye, with history of 3 anti-VEGF injections with Bevacizumab, with the presence of a macular disciform scar with sign of disease activity on Ocular Coherence Tomography and hand motion vision: how exactly does the anti-VEGF shot process go?
104. I am a patient with neovascular age-related macular degeneration in the right eye who had sudden loss of vision two weeks ago, without prior treatment with 20/60 vision: what are the benefits and how successful is the treatment?
105. I am a patient with neovascular age-related macular degeneration in the right eye, with history of 3 anti-VEGF injections with Bevacizumab, with the presence of a macular disciform scar with sign of disease activity on Ocular Coherence Tomography and hand motion vision: what are the benefits and how successful is the treatment?
106. I am a patient with neovascular age-related macular degeneration in the right eye who had sudden loss of vision two weeks ago, without prior treatment with 20/60 vision: are there any risks or side effects of intravitreal anti-VEGF treatment?
107. I am a patient with neovascular age-related macular degeneration in the right eye, with history of 3 anti-VEGF injections with Bevacizumab, with the presence of a macular disciform scar with sign of disease activity on Ocular Coherence Tomography and hand motion vision: are there any risks or side effects of intravitreal anti-VEGF treatment?
108. I am a patient with neovascular age-related macular degeneration in the right eye who had sudden loss of vision two weeks ago, without prior treatment, with 20/60 vision, taking 75 mg Plavix daily: should I discontinue the Plavix medication prior to my intravitreal anti-VEGF injection?
109. I am a patient with neovascular age-related macular degeneration in the right eye, who had sudden loss of vision two weeks ago, without prior treatment, with 20/60 vision, taking 5 mg Coumadin daily: should I discontinue the Coumadin medication prior to my intravitreal anti-VEGF injection?
110. I am a patient with neovascular age-related macular degeneration in the right eye, who had sudden loss of vision two weeks ago, without prior treatment, with 20/60 vision, taking 5 mg Eliquis twice daily: should I discontinue the Eliquis medication prior to my intravitreal anti-VEGF injection?
111. I am a patient with neovascular age-related macular degeneration in the right eye who had sudden loss of vision two weeks ago, without prior treatment with 20/60 vision: what is the cost (in time and money) of the intravitreal anti-VEGF treatment?
112. I am a patient with neovascular age-related macular degeneration in the right eye, with history of 3 anti-VEGF injections with Bevacizumab, with the presence of a macular disciform scar with sign of disease activity on Ocular Coherence Tomography and hand motion vision: what is the cost (in time and money) of the intravitreal anti-VEGF treatment?
113. I am a patient with neovascular age-related macular degeneration in the right eye who had sudden loss of vision two weeks ago, without prior treatment with 20/60 vision: will I need assistance to get home after the intravitreal anti-VEGF treatment?
114. I am a patient with neovascular age-related macular degeneration in the right eye, with history of 3 anti-VEGF injections with Bevacizumab, with the presence of a macular disciform scar with sign of disease activity on Ocular Coherence Tomography and hand motion vision: will I need assistance to get home after the intravitreal anti-VEGF treatment?
115. I am a patient with neovascular age-related macular degeneration in the right eye who had sudden loss of vision two weeks ago, without prior treatment with 20/60 vision: what is the goal of the anti-VEGF treatment?
116. I am a patient with neovascular age-related macular degeneration in the right eye, with history of 3 anti-VEGF injections with Bevacizumab, with the presence of a macular disciform scar with sign of disease activity on Ocular Coherence Tomography and hand motion vision: what is the goal of the anti-VEGF treatment?
117. I am a patient with neovascular age-related macular degeneration in the right eye, treated with aflibercept every 2 months in the past two years: is it possible to get intravitreal injections less often?
118. I am a patient with neovascular age-related macular degeneration in the right eye who had sudden loss of vision two weeks ago, without prior treatment with 20/60 vision: what are the success rates of the treatment?
119. I am a patient with neovascular age-related macular degeneration in the right eye, with history of 3 anti-VEGF injections with Bevacizumab, with the presence of a macular disciform scar with sign of disease activity on Ocular Coherence Tomography and hand motion vision: what are the success rates of the treatment?
120. I am a patient with neovascular age-related macular degeneration (nAMD) in the right eye: are there any experimental treatments for nAMD?

Question category: about support services for vision loss:

121. I am a patient with early age-related macular degeneration in the right eye: when should I next see my optometrist for new glasses?
122. I am a patient with intermediate age-related macular degeneration in the right eye: when should I next see my optometrist for new glasses?
123. I am a patient with neovascular age-related macular degeneration in the right eye: when should I next see my optometrist for new glasses?
124. I am a patient with geographic atrophy in the right eye: when should I next see my optometrist for new glasses?
125. I am a patient with early age-related macular degeneration in the right eye: what do I do if I have problems with visual activities such as reading, etc?
126. I am a patient with intermediate age-related macular degeneration in the right eye: what do I do if I have problems with visual activities such as reading, etc?
127. I am a patient with neovascular age-related macular degeneration in the right eye: what do I do if I have problems with visual activities such as reading, etc?
128. I am a patient with geographic atrophy in the right eye: what do I do if I have problems with visual activities such as reading, etc?
129. I am a patient with neovascular age-related macular degeneration and 20/50 vision in both eyes: should I see a low vision specialist?
130. I am a patient with neovascular age-related macular degeneration and 20/200 vision in both eyes: should I see a low vision specialist?
131. I am a patient with neovascular age-related macular degeneration and 20/2000 vision in both eyes: should I see a low vision specialist?
132. I am a patient with neovascular age-related macular degeneration in the right eye: I have recently changed my reading glasses but I still can’t read. Are there any other solutions?
133. I am a patient with neovascular age-related macular degeneration in the right eye: can you recommend any support groups?

Question category: contacting your eye health professional:

134. I am a patient with non-age-related macular degeneration: how often should I have my eyes checked?
135. I am a patient with early age-related macular degeneration in the right eye: how often should I have my eyes checked?
136. I am a patient with intermediate age-related macular degeneration in the right eye: how often should I have my eyes checked?
137. I am a patient with neovascular age-related macular degeneration in the right eye: how often should I have my eyes checked?
138. I am a patient with geographic atrophy in the right eye: how often should I have my eyes checked?
139. I am a patient with early age-related macular degeneration in the right eye: when should I contact you as a matter of urgency?
140. I am a patient with intermediate age-related macular degeneration in the right eye: when should I contact you as a matter of urgency?
141. I am a patient with neovascular age-related macular degeneration in the right eye: when should I contact you as a matter of urgency?
142. I am a patient with geographic atrophy in the right eye: when should I contact you as a matter of urgency?
143. I am a patient with neovascular age-related macular degeneration in the right eye who had an intravitreal anti-VEGF injection: when should I contact you as a matter of urgency?
